# Supplementary material for: Chromothripsis during telomere crisis is independent of NHEJ, and consistent with a replicative origin
Source: Genome Res. 2019 May;29(5):737–49. doi: 10.1101/gr.240705.118 (PMC6499312; doi:10.1101/gr.240705.118)
Supplement: Supplemental Material [file supp_gr.240705.118_Supplemental_file_1.zip › contigs/annotated_contigs/DB57/contig.2.DB57_length_294_mean_cov_33.7482993197.docx]

**DB57_length_294_mean_cov_33.7482993197**

```
GCGGCTAGGAGGAAAAAAACGAGTCGGGGA|GGGGGGGGAGGAGGGGGGGGGGGAGGGGGGGGGGGGGGGGG|GAG|AGAGAGAGAGAG
                               >chr2:33141358-33141402 + E=4e-06        >chrUn_gl00022
AGAGAGAGAGAGAGAGAGAGAGAGAAGTAAAACCAAACACCACCTCCTTGACCTGAGTCGGGGGTTTCTGGCCTTTTGGGAGAACGTTG
0:130057-130224 + E=3e-71
AGCGACAATGCAGTATTTGGGCCCGTTCTTTTTTTTCTTCTTCTTCTTTTCTTTCTTTTTTTT|GGTCTTAGTCTCTCTGTTTTTTTTA

CACTGTCTGATTTGCTTTGGTGCTGTCTTGG
```
